# Marine biogenic emissions of benzene and toluene and their contribution to secondary organic aerosols over the polar oceans

**DOI:** 10.1126/sciadv.add9031

**Published:** 2023-01-27

**Authors:** Charel Wohl, Qinyi Li, Carlos A. Cuevas, Rafael P. Fernandez, Mingxi Yang, Alfonso Saiz-Lopez, Rafel Simó

**Affiliations:** ^1^ Department of Marine Biology and Oceanography, Institut de Ciències del Mar, ICM-CSIC, Barcelona 08003, Catalonia, Spain.; ^2^ Plymouth Marine Laboratory, Plymouth PL1 3DH, UK.; ^3^ Department of Atmospheric Chemistry and Climate, Institute of Physical Chemistry Rocasolano, IQFR-CSIC, Madrid 28006, Spain.; ^4^ Institute for Interdisciplinary Science (ICB), National Research Council (CONICET), FCEN-UNCuyo, Mendoza 5500, Argentina.

## Abstract

Reactive trace gas emissions from the polar oceans are poorly characterized, even though their effects on atmospheric chemistry and aerosol formation are crucial for assessing current and preindustrial aerosol forcing on climate. Here, we present seawater and atmospheric measurements of benzene and toluene, two gases typically associated with pollution, in the remote Southern Ocean and the Arctic marginal ice zone. Their distribution suggests a marine biogenic source. Calculated emission fluxes were 0.023 ± 0.030 (benzene) and 0.039 ± 0.036 (toluene) and 0.023 ± 0.028 (benzene) and 0.034 ± 0.041 (toluene) μmol m^−2^ day^−1^ for the Southern Ocean and the Arctic, respectively. Including these average emissions in a chemistry-climate model increased secondary organic aerosol mass concentrations only by 0.1% over the Arctic but by 7.7% over the Southern Ocean, with transient episodes of up to 77.3%. Climate models should consider the hitherto overlooked emissions of benzene and toluene from the polar oceans.

## Teaser

Biogenic benzene and toluene emissions from the polar oceans enhance atmospheric secondary organic aerosol mass.

## INTRODUCTION

The microbiota of the world’s oceans produce a plethora of organic gases, leading to ocean emissions (*1*–*4*). Such emissions are particularly important for atmospheric chemistry in the polar regions that are relatively pristine and far away from terrestrial sources (*5*, *6*). Among organic gases, nonmethane hydrocarbons can act as a sink for OH in marine air (*7*, *8*) and can contribute substantially to aerosol mass (*9*–*11*) and new particle formation (*5*). Accurately quantifying natural marine aerosol particularly in the polar regions is crucial to estimate aerosol climate forcing (*9*, *12*, *13*), especially since secondary organic aerosols (SOAs) have been suggested to exert a strong effect on cloud formation (*14*). The term SOA refers to the organic fraction of aerosol derived from atmospherically oxidized organic precursor compounds. Global models currently hugely underestimate marine aerosol (*15*, *16*) and parameters such as aerosol optical depth (*17*) or aerosol concentrations (*18*) in regions where anthropogenic influence is lowest. Studying the pristine ocean atmosphere allows understanding and setting of the preindustrial (i.e., pristine) baseline of SOA precursors, which is critical for modeling assessments of anthropogenic forcing (*19*, *20*).

Solving the aerosol underestimate by models requires quantifying the total marine emissions of aerosol-forming organic gases and accurately predicting their paths to SOA formation. Revealing the identity of these gases is crucial as well because the efficiency with which organic gases are oxidized into SOA components varies largely across compounds. Chamber studies generally show that the aerosol-forming yield of to-date well-studied marine biological gases is rather low—2 to 7% for dimethyl sulfide (DMS) (*21*), around 2% for isoprene (*22*), and 1 to 68% for monoterpenes depending on the species (*23*). In contrast, the SOA-forming yield is much higher for benzene (36%) and toluene (30%), especially in low-NO*_x_* regimes (*24*). Benzene and toluene are traditionally associated with anthropogenic emissions, the largest sources being coal or petroleum combustion, crude oil processing, and solvent use followed by biomass burning (*25*). Their largest sink in the atmosphere is the oxidation by rapid reaction with OH (*25*). Benzene and toluene have been measured in air of the remote polar and temperate oceans (*26*, *27*), suggesting that they are common and widespread organic species in the atmosphere. It is unclear whether the oceans act as a source or a sink of benzene and toluene, especially in natural environments. If occurring, then sea-to-air fluxes (ocean source) of benzene and toluene to the remote marine atmosphere would be particularly impactful owing to their high SOA formation potential and rapid reaction rate with OH. Guo *et al.* (*28*) found traces of aromatic (i.e., benzene and toluene) oxidation products in marine organic aerosol in the northwest Pacific.

Recently, Giorio *et al.* (*29*) (see the Supporting Information thereof) observed toluene in the headspace of seawater and ocean foam samples from the Benguela upwelling system. They evoked a biological source and a sea-to-air flux to explain the high toluene air mole fractions observed in marine air (*29*). Rocco *et al.* (*30*) suggested a phytoplankton source of benzene and toluene in the surface ocean. They used mesocosm and phytoplankton culture experiments to show that phytoplankton species produce benzene and toluene at variable rates. Misztal *et al.* (*31*) also used mesocosm experiments to suggest a diurnally varying source of toluene from the coccolithophore microalga *Emiliania huxleyi*. There also exists some evidence for toluene production by the coral microalgal symbionts Symbiodiniaceae and two members of its core microbiome (*32*). Toluene has been found to be produced by 20 bacterial genera (*33*) and by bacteria isolated from surface seawater in Antarctica (*34*). Korpi *et al.* (*35*) listed benzene and toluene as compounds frequently reported as microbial volatile organic compounds (VOCs). While experiments show that benzene and toluene can be produced by marine biota, in situ measurements are needed to quantify the magnitude of their air-sea flux and the atmospheric importance thereof.

Air-sea benzene and toluene fluxes can be estimated using the bulk two-layer model (*36*), which requires measured concentrations of benzene and toluene in the surface ocean and lower atmosphere. Ambient air measurements of benzene and toluene have become very common, especially since the introduction of proton transfer reaction–mass spectrometers (PTR-MSs) (*26*, *27*, *37*, *38*). However, to our knowledge, there have been very few reported concentrations of benzene and toluene dissolved in seawater. Only Sauer (*39*) reported 64 to 192 pmol dm^−3^ of benzene and 32 to 108 pmol dm^−3^ of toluene in unpolluted surface seawater of the Gulf of Mexico. There exist no measurements alongside biological proxies in the water column at different depths, which could give important clues to their sources and sinks in the ocean. With anthropogenic activity likely to increase in the changing polar regions, seawater measurements of benzene and toluene are timely and represent a useful benchmark. Rocco *et al.* (*30*) performed benzene and toluene flux observations in the Southern Ocean; however, their flux estimates were restricted to a few episodes and relied upon the indirect nocturnal accumulation method (*30*, *40*). Such observations of episodic outgassing from the ocean are not sufficient to ascertain whether the ocean is a net source of benzene and toluene, given the variety of other sources to the global atmosphere, including shipping fuel combustion.

Here, we present benzene and toluene measurements in seawater and ambient air in the open Southern Ocean and the Arctic marginal sea ice zone. Our unique seawater measurements from both polar oceans include high-resolution surface underway measurements and depth profiles, which are compared to chlorophyll fluorescence and density profiles to explore the possible marine sources of benzene and toluene. The high-resolution measurements at both sides of the air-sea interface are used to calculate the oceanic saturation and the net fluxes at high resolution using the two-layer bulk flux method. These oceanic emission fluxes are incorporated in a global chemistry-climate model, the Community Atmospheric Model with Chemistry [CAM-Chem (*41*)], to assess the atmospheric implications of ocean benzene and toluene outgassing over the polar oceans. We find that benzene and toluene emission fluxes notably increase the modeled SOA, especially over the remote Southern Ocean.

## RESULTS

### Southern Ocean observations

#### Depth profiles

**Fig. 1.**
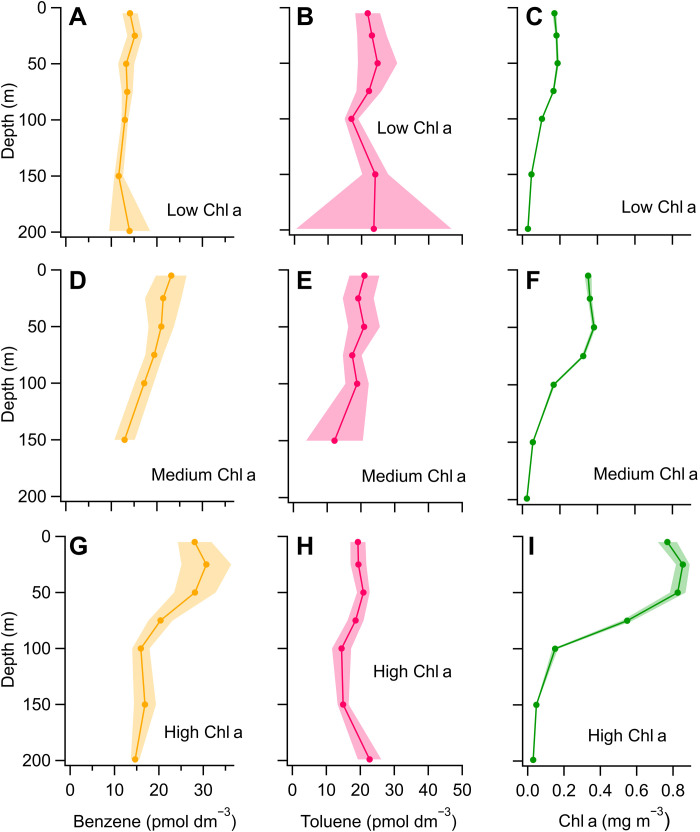
Concentrations measured in the water column of the Southern Ocean. This overview plot displays the averaged casts of benzene (**A**, **D**, and **G**), toluene (**B**, **E**, and **H**), and Chl a (**C**, **F**, and **I**) from the Southern Ocean cruise. The casts are grouped by surface Chl a concentration (low, medium, and high Chl a) and depth bin averaged. The shaded area indicates the standard error of each depth bin. Depth bins containing less than two data points are not shown to avoid bias. Standard error in panel C is very small. The number of individual casts in each Chl a group is 11 for low Chl a, 10 for medium Chl a, and 7 for high Chl a.

A total of 28 CTD (conductivity/temperature/depth) stations were vertically profiled for benzene and toluene in the Southern Ocean. For the ease of visualization and analysis, the CTD stations were classified by surface Chl a (chlorophyll a) concentration: low Chl a, 0 to 0.2 mg m^−3^; medium Chl a, 0.2 to 0.5 mg m^−3^; and high Chl a, 0.5 to 1 mg m^−3^. Higher Chl a concentrations are indicative of high phytoplankton activity. These thresholds were chosen as they corresponded to patterns in benzene and toluene depth profile distributions and reflect the distribution of Chl a in the Southern Ocean (*42*). The highest surface Chl a concentration measured during the cruise was 1 mg m^−3^. Grouped stations were averaged in depth bins to help tease out patterns (Fig. 1). Cruise track with CTD sampling locations (fig. S1) and individual vertical profiles of benzene, toluene, Chl a, and seawater density are shown in the Supplementary Materials (figs. S2 and S3), while additional details regarding the Chl a data, analytical chemistry, and measurement uncertainty are provided in the Supplementary Text.

The depth profiles reveal that benzene concentrations were generally higher in the top 75 m, along with elevated Chl a concentrations. The benzene profiles displayed concentrations of around 10 pmol dm^−3^ in the upper 200 m in low–Chl a casts and, on average, around 20 and 30 pmol dm^−3^ in the upper 75 m in the medium– and high–Chl a casts, respectively. Chl a and benzene depth profiles showed remarkable covariation with depth across Chl a regimes. Conversely, toluene concentrations were not always the highest in the top 75 m, and their profile shape and mean concentrations did not always follow the distribution of Chl a. However, higher toluene concentrations near the surface were commonly observed in casts with high Chl a. The absence of a clear pattern with depth is probably due to toluene origin not only in phytoplankton but also in bacteria (*33*, *34*), which are not related to Chl a, as well as toluene’s short lifetime in seawater (*43*, *44*) that prevents accumulation. These depth profiles support distinct biological sources of benzene and toluene in seawater.

#### Underway surface concentrations and flux estimates

**Fig. 2.**
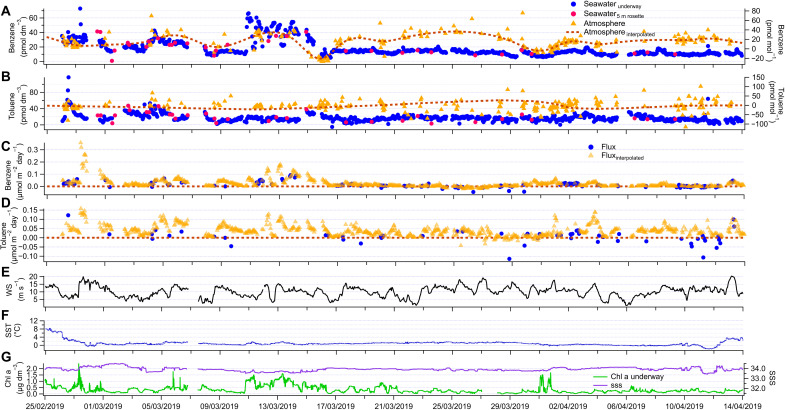
Underway measurements from the Southern Ocean. Hourly underway surface seawater concentrations and atmospheric mole fractions of benzene and toluene in (**A**) and (**B**), respectively. Interpolated air mole fractions are also shown in (A) and (B). The calculated sea-to-air fluxes are shown in (**C**) and (**D**). Positive fluxes indicate ocean outgassing, i.e., sea-to-air fluxes. The other plots show the wind speed (WS) (**E**), underway sea surface temperature (SST) (**F**), and Chl a and surface seawater salinity (SSS) (**G**).

Ambient air, underway surface seawater measurements, and calculated air-sea fluxes of benzene and toluene are shown in Fig. 2. Positive fluxes indicate oceanic outgassing, i.e., sea-to-air flux. Interruptions in the air measurements were largely due to ship stack contamination. Fluxes were computed in two ways, using either measured air mole fractions or interpolated air measurements. Both sets of fluxes are weighted by the number of data points to avoid bias in the reported cruise means.

Figure 2 shows that frequent measurements in discrete samples taken at a depth of 5 m from CTD casts compared well (within measurement noise) with concurrent measurements from the underway intake system. Considering the measurements from the CTD cast to be clean, i.e., unaffected by the sampling method, this confirms that the ship’s underway intake system did not contaminate our measurements. Mean underway surface seawater benzene and toluene concentrations were very similar (cruise means ± SD; benzene, 17 ± 11 pmol dm^−3^; toluene, 17 ± 9 pmol dm^−3^), and both displayed a large range: for benzene, from the limit of detection (LOD) to 73 pmol dm^−3^, and for toluene, from 5 to 72 pmol dm^−3^. To put these seawater concentrations into context, another climate-relevant volatile such as isoprene was also present in seawater at such low concentrations [cruise mean, 13.3 pmol dm^−3^ (*45*)]. Previous mesocosm experiments (*31*) have suggested a diurnally varying source of benzene and toluene in the ocean (*31*). However, diurnal variability is not obvious in our data (fig. S4). Instead, we observed sporadic episodes of very high seawater benzene and toluene concentrations on top of relatively constant background levels. This is a very characteristic distribution for compounds produced by biological processes, as is the case, for example, for DMS (*2*) and isoprene (*46*). The median seawater concentration of benzene (13 pmol dm^−3^) and toluene (15 pmol dm^−3^) was lower than the mean, which illustrates a greater skewness for benzene than for toluene. Despite similar mean concentrations, benzene and toluene usually did not peak at the same time. For example, benzene concentrations in seawater were very high in an area of high Chl a concentration sampled around 13 March 2019, while toluene concentrations in this area were near the cruise average. Consequently, benzene and toluene underway seawater concentrations correlated significantly but weakly during this cruise track (*R* = 0.07; *P* < 0.001; *n* = 885) (fig. S5), suggesting different source and sink strengths for the two compounds. Benzene correlated significantly but weakly with Chl a (*R* = 0.34; *P* < 0.001; *n* = 859) and strongly with DMS (*R* = 0.70; *P* < 0.001; *n* = 868), while toluene did not correlate with either. The correlation of benzene with Chl a is consistent with the findings from the depth profiles. Toluene did correlate weakly with isoprene (*R* = 0.08; *P* < 0.001; *n* = 825). Overall, significant correlations with a phytoplankton proxy (Chl a) and/or other biogenic trace gases (DMS and isoprene) support a biological source for benzene and toluene in the upper ocean.

Cruise mean ambient air benzene mole fractions were lower than typically observed at terrestrial or coastal sites (benzene, 14 ± 17 pmol mol^−1^) (*25*, *29*), while toluene mole fractions were essentially near zero and below the LOD for most of the cruise track. Such low ambient air mole fractions over the Southern Ocean are similar to observations by Guérette e*t al.* (*38*) and Rocco *et al.* (*30*), who also observed less than 20 pmol mol^−1^ of benzene and toluene on average over the Southern Ocean. Our observations of higher concentrations of benzene compared to toluene are likely related to the faster reaction with OH and shorter atmospheric lifetime of toluene (*24*). Low and homogeneous mole fractions of benzene and toluene and the absence of a significant correlation (*R* = 0.01; *P* = 0.1; *n* = 237) between benzene and toluene mole fractions suggest that the concentrations measured during this cruise were largely produced by local and diffuse sources, e.g., many episodes of oceanic outgassing rapidly mixed by winds, and not by transport of polluted air masses.

During this cruise, the fluxes of benzene and toluene were almost always from the ocean to the atmosphere. The cruise mean saturation (an indication of the thermodynamic forcing and direction of the flux; here, a saturation above 100% indicates outgassing) was 192 ± 121% for benzene and 173 ± 106% for toluene, while the cruise mean fluxes were 0.023 ± 0.030 μmol m^−2^ day^−1^ for benzene and 0.039 ± 0.036 μmol m^−2^ day^−1^ for toluene. There was a large range in the benzene and toluene fluxes, with a maximum of 0.358 μmol m^−2^ day^−1^ for benzene and 0.158 μmol m^−2^ day^−1^ for toluene. To put these emissions into context, the mean benzene and toluene fluxes out of the ocean were comparable (i.e., within 0.007 μmol m^−2^ day^−1^) to the mean isoprene flux measured during the same cruise (*45*). These benzene and toluene fluxes are also in the same order of magnitude (i.e., within 0.012 μmol m^−2^ day^−1^) as the mean monoterpene fluxes reported from the northwest Atlantic (*47*). This illustrates that, on a molar-per-area basis, benzene and toluene fluxes rival emissions by other hitherto better known marine biological gases. We observe lower median fluxes of benzene (0.012 μmol m^−2^ day^−1^) and toluene (0.034 μmol m^−2^ day^−1^) compared to the mean, which illustrates skewedness of the data distribution and larger outgassing events influencing the mean. Rocco *et al.* (*30*) calculated emissions of 1.3 μmol m^−2^ day^−1^ (=1.2 ng m^−2^ s^−1^) of benzene and 3.5 μmol m^−2^ day^−1^ (=3.7 ng m^−2^ s^−1^) of toluene. This is more than 60 times larger than our mean and 3 (benzene) and 22 (toluene) times larger than our highest outgassing fluxes. It is worth noting that the flux estimates from Rocco *et al.* (*30*) are based on three nights only and were calculated using the nocturnal accumulation method. This method assumes that nighttime losses from reaction with OH are negligible and that ocean emissions accumulate overnight in a well-mixed marine boundary layer. Any nighttime increase [which have only been detected reliably for three nights in their works (*30*)] is assumed to be due to ocean outgassing. However, this is not necessarily a given for atmospheric benzene and toluene because of the existence of continental sources of these compounds. This method can only be applied to nights when there was substantial outgassing, which could lead to an overestimate of the true mean flux. The nocturnal accumulation method has thus far only been used for DMS (*40*) and methanethiol (*48*) and only validated for DMS, two gases that are well known to be consistently supersaturated in the ocean and have essentially only marine sources. The flux estimates from the cruises presented here represent an average based on a large number of data points and covering a large geographical area using a well-established method.

### Canadian Arctic observations

#### Depth profiles

**Fig. 3.**
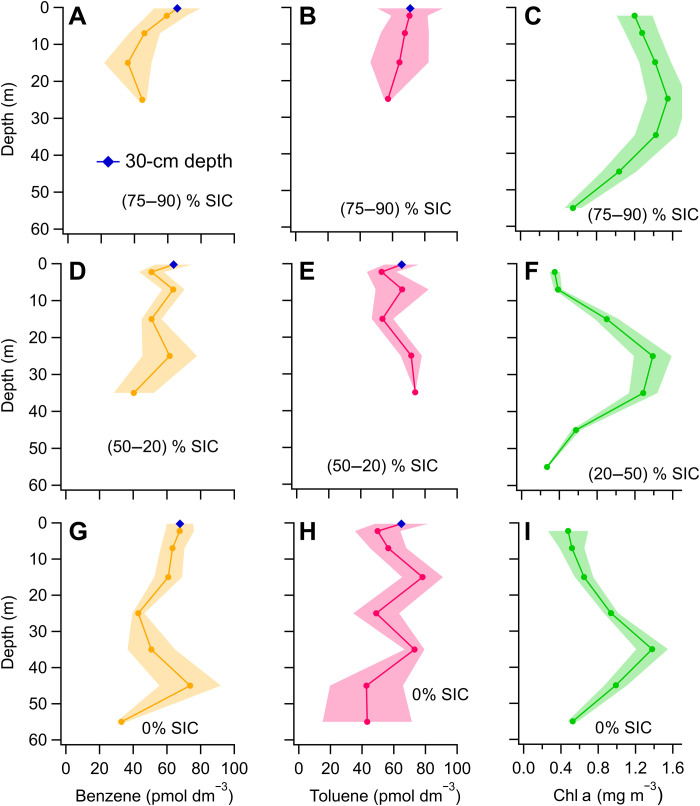
Concentrations measured in the water column of the Arctic sea ice zone. Depth bin–averaged casts of benzene (**A**, **D**, and **G**), toluene (**B**, **E**, and **H**), and Chl a (**C**, **F**, and **I**) from the Arctic cruise. The casts are grouped by SIC and depth bin averaged. The shaded area indicates the standard error of each depth bin. Depth bins containing less than two data points are not shown to avoid bias. The number of individual casts in each SIC group is 4 for 75 to 90%, 7 for 20 to 50%, and 13 for 0% SIC.

A total of 24 depth profiles were measured during this deployment. In the marginal ice zone in summer and spring, the sea-ice cover (SIC) controls the biogeochemistry and the physical structure of the water column, including the distribution of Chl a and density (*49*) and bacterial activity (*50*). Thus, exactly as in the work of Wohl *et al.* (*51*), vertical profiles were classified by SIC at the time of sampling (Fig. 3). Details of the Chl a measurements (*52*), analytical chemistry, measurement noise, and cruise track are presented in the Supplementary Text.

The individual depth profiles of benzene and toluene showed less identifiable patterns than those in the Southern Ocean (figs. S6 and S7). Nevertheless, depth bin averaging allowed us to visually tease out some trends clearer (Fig. 3). Within the 75 to 90% SIC bin (near-full SIC), benzene and toluene concentrations gradually decreased from the surface to 20 to 30 m. At partial SIC (20 to 50% SIC), benzene and toluene were mixed more homogeneously within the 40 m near the surface. Under ice-free conditions (0% SIC), subsurface peaks in benzene and toluene occurred, which were often situated around a subsurface peak in Chl a concentration. In the Arctic, a substantial amount of biological activity occurs below the surface at this Chl a peak (*53*). Thus, subsurface peaks of benzene and toluene that colocated with Chl a support a biological source. Other biogenic gases, such as DMS and isoprene, also displayed higher concentrations at the depth of peak Chl a concentration compared to 5 m below the surface (*51*). About 20% of the stations displayed higher concentrations at 30 cm than at 2 m. The concentration increase often coincided with lower seawater density (figs. S5 and S6) and suggests fine-scale vertical variability in the processes controlling the concentrations of benzene and toluene, similar to other biogenic gases during this cruise (*51*). Overall, these measurements support distinct biological sources for benzene and toluene in the Arctic Ocean related to Chl a, although the precise factors controlling their distribution are far from clear.

#### Underway concentrations and flux estimates

**Fig. 4.**
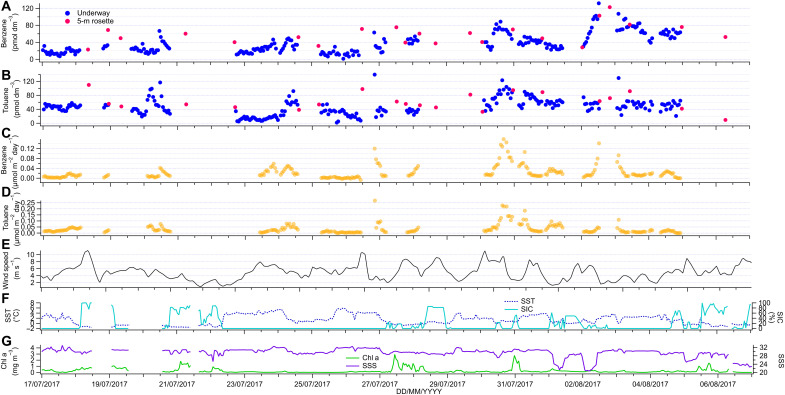
Underway measurements from the Arctic marginal sea-ice zone. Benzene and toluene underway surface seawater concentrations (**A** and **B**) and air-sea fluxes (**C** and **D**). Positive fluxes indicate ocean outgassing, i.e., sea-to-air flux. The other plots show the wind speed (**E**), underway SST and SIC (AMSR2) (**F**), and Chl a and SSS (**G**).

Underway concentration measurements and air-sea flux estimates of benzene and toluene in the Canadian Arctic marginal sea-ice zone during boreal summer are shown in Fig. 4. Continuous underway and 5-m discrete measurements from CTD casts agreed well within measurement uncertainty, confirming that the research ship’s underway seawater intake system did not contaminate our measurements. Mean seawater concentrations were two times higher for benzene (37 ± 24 pmol dm^−3^) and three times higher for toluene (46 ± 24 pmol dm^−3^) than those in the Southern Ocean cruise. This could be due to either higher biological activity (it was earlier in the productive season) or larger anthropogenic influence and thus higher contribution of anthropogenic benzene and toluene to observed seawater concentrations. Because of the short residence times of benzene and toluene in unpolluted seawater [less than 1 day (*44*)], we expect any anthropogenic influence to be due to local sources. Similar to the cruise in the Southern Ocean, concentrations displayed a wide range from near the LOD to up to 131 and 139 pmol dm^−3^ for benzene and toluene, respectively. Also, similar to the Southern Ocean, the median benzene concentration (30 pmol dm^−3^) was lower than the mean, although in contrast, toluene median concentration was the same as the mean. Skewed data distribution of peak concentrations among an apparently constant background is typical for biologically produced compounds. There was no significant correlation between Chl a and benzene (0 < *R* < 0.01; *P* = 0.53; *n* = 219) or toluene (0 < *R* < 0.02; *P* = 0.058; *n* = 223). The depth profiles suggest that the lack of a correlation with Chl a is probably because much of the biological activity and production of benzene and toluene occurred at depths below 5 m, while the underway measurements were taken at 3 to 4 m. Benzene and toluene also did not correlate with isoprene, DMS, or SIC. In comparison to the Southern Ocean, benzene and toluene correlate with a higher *R* value in the Canadian Arctic (*R* = 0.21; *P* < 0.001; *n* = 245) (fig. S8), suggesting that in the Arctic, seawater concentrations are more influenced by local anthropogenic sources. Overall, the underway surface ocean measurements further support that at least part of the benzene and toluene had a biological origin, potentially with contributions from local pollution.

Atmospheric mole fractions of benzene and toluene were not measured on the Arctic cruise. To estimate air-sea flux and mean saturation, we used the previous air measurements of these compounds at a similar location (western Canadian Arctic) and similar time of year (August to September) by Sjostedt *et al.* (*37*) (13 pmol mol^−1^ of benzene and 4 pmol mol^−1^ of toluene). This is comparable to a more recent study by Pernov *et al.* (*54*), who measured 27 pmol mol^−1^ of benzene near the coast in Northeast Greenland. Using the measurements from the work of Sjostedt *et al.* (*37*), saturation and fluxes were calculated and are presented in Fig. 4. The ocean was highly supersaturated in benzene (610 ± 386%) and toluene (2335 ± 1175%), and thus, uncertainty in the air mole fractions should not affect the flux very much, similarly to isoprene (*55*). In table S1, we show alternative fluxes and saturations calculated with air mole fractions modeled by Cabrera-Perez *et al.* (*25*), which are higher than those measured by Sjostedt *et al.* (*37*). The results illustrate that the choice of the air mole fraction does not change the conclusion that the Arctic Ocean was consistently outgassing benzene and toluene. Cruise mean fluxes were 0.023 ± 0.028 μmol m^−2^ day^−1^ for benzene and 0.034 ± 0.041 μmol m^−2^ day^−1^ for toluene. Surface ocean concentrations and supersaturations were much higher in the Arctic than in the Southern Ocean. However, the mean fluxes from the two cruises were similar. This is due to the lower estimated air-sea transfer velocities in the Arctic, which resulted from lower wind speeds and sea ice acting as a barrier to air-sea exchange in our calculation. Benzene and toluene fluxes displayed a lower median than mean flux (benzene median flux, 0.014 μmol m^−2^ day^−1^; toluene median flux, 0.020 μmol m^−2^ day^−1^). Therefore, reported mean fluxes were influenced by strong outgassing episodes that coincided with above-average seawater concentrations, high winds, and low SIC, e.g., on 31 July and 1 August. The highest hourly measured outgassing flux was 0.158 μmol m^−2^ day^−1^ for benzene and 0.268 μmol m^−2^ day^−1^ for toluene. Similar to the cruise in the Southern Ocean, the mean emissions of benzene and toluene from this cruise in the Arctic marginal ice zone are comparable (within 0.024 μmol m^−2^ day^−1^) to the isoprene flux from the same deployment (*51*).

Although our flux calculations were not very sensitive to a range of realistic atmospheric mole fractions, could an increased anthropogenic activity in the Arctic reverse the direction of the benzene and toluene fluxes? If we assumed around 50 pmol mol^−1^ of benzene and 30 pmol mol^−1^ of toluene in the marine atmosphere as modeled for the busy shipping corridors of the North Atlantic and North Pacific (*25*), the estimated mean saturations would be 159 and 311%, and the mean fluxes would be 0.011 and 0.026 μmol m^−2^ day^−1^ for benzene and toluene, respectively. Assuming that seawater concentrations remain unaffected by shipping, this suggests that even if anthropogenic activity largely increased in the Arctic, benzene and toluene are likely still to be emitted to the atmosphere from the ocean. Ambient air mole fractions above 79 pmol mol^−1^ of benzene and 91 pmol mol^−1^ of toluene would be required to lead to ocean uptake in the Arctic summer (considering the seawater solubilities and the mean benzene and toluene seawater concentrations from this cruise). Such high atmospheric mole fractions are only predicted for densely populated areas (*25*).

### Atmospheric effects of oceanic benzene and toluene emissions

The effects of these ocean emissions of biogenic benzene and toluene on atmospheric chemistry were assessed using a global chemistry-climate model [CAM-Chem (*41*)]. Table 1 shows simulated atmospheric mole fractions of benzene, toluene, and OH in the three simulation cases (no BT, avg BT, and max BT; table S2), along with the changes between cases. Adding our mean measured benzene and toluene ocean flux to the model increases benzene and toluene atmospheric mole fractions by 1.7 to 3.2 pmol mol^−1^ with respect to the no BT case where there is no oceanic efflux, and polar airborne benzene and toluene result from long-distance transport alone. Because of the low simulated background concentrations, the contribution of oceanic emissions represents a 12% (benzene) and 100% (toluene) increase in the Arctic and as much as a 3-fold (benzene) and 30-fold (toluene) increase in the Southern Ocean. The model results compare well with our Southern Ocean observations of 14 ± 17 pmol mol^−1^ of benzene, while our measured toluene mole fractions were below the LOD. The air mole fractions modeled for the Arctic in the no BT case are within 7 pmol mol^−1^ of the ones used to compute the flux and saturations, which is reassuring.

**Table 1.** Impact of benzene and toluene ocean emissions on atmospheric composition and OH. Sampling months were February to March 2019 for the Southern Ocean and July to August 2017 for the Arctic. The three model runs were as follows: no BT, no ocean benzene/toluene fluxes; avg BT, mean benzene/toluene ocean fluxes; and max BT, the highest measured benzene/toluene fluxes (table S2). “absdiff,” absolute difference; “reldiff,” relative difference or relative change.

		No BT	Avg BT			Max BT		
		Mole fractions (pmol mol^−1^)	Mole fractions (pmol mol^−1^)	Absdiff (pmol mol^−1^)	Reldiff (%)	Mole fractions (pmol mol^−1^)	Absdiff (pmol mol^−1^)	Reldiff (%)
Arctic	Benzene	19.7	22.1	2.4	12	34.8	15.1	77
	Toluene	1.7	3.4	1.7	100	15.7	14.0	824
	OH	0.06	0.06	0	<0.01	0.06	0	<0.02
Antarctic	Benzene	0.9	4.1	3.2	355	48.3	47.4	5,059
	Toluene	0.1	3.1	3.0	3000	12.1	12.0	12,000
	OH	0.008	0.008	0	<0.01	0.008	0	<0.01

Despite their rapid reaction with OH, because of their comparatively low fluxes, the oceanic benzene and toluene emissions do not notably affect modeled OH concentrations, which decrease by less than 0.02% even when the cruise-maximum emissions are considered. Thus, OH sinks other than benzene and toluene prevail (*7*, *8*). A better assessment of the overall impact of benzene and toluene on OH would require a more complete emission inventory of aromatics in the model. For example, using a comprehensive list of annual anthropogenic and biomass burning emissions, Taraborrelli *et al.* (*56*) found that aromatics decrease model OH concentrations over the ocean by 2 to 5%.

**Fig. 5.**
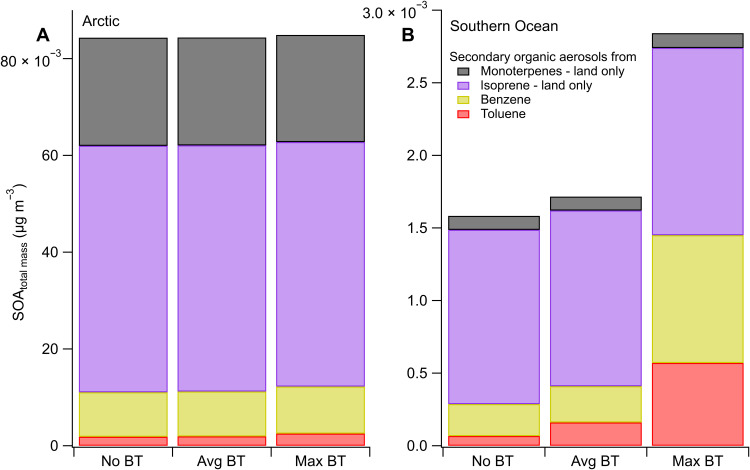
Modeled impact of benzene and toluene fluxes on SOA. Shown are the modeled impact of benzene and toluene ocean emission fluxes on SOA mass concentrations in (**A**) the Arctic (July to August 2017) and (**B**) the Southern Ocean (February to April 2019). Three model runs are presented that differ by their ocean benzene and toluene ocean emissions; “no BT,” no ocean benzene/toluene fluxes; “avg BT,” mean benzene/toluene ocean fluxes; “max BT,” the highest measured benzene/toluene fluxes.

However, the inclusion of our mean and maximum benzene and toluene emission fluxes increases benzene- and toluene-derived SOA by ~1 and 6.3% in the Arctic. Over the Southern Ocean, the increases in benzene- and toluene-derived SOA due to including oceanic emissions are 12.8 and 139.8% (Fig. 5). Note that the simulated background SOA mass concentrations in the Arctic are more than 25 times those in the Southern Ocean. Most of benzene and toluene in the Arctic that forms SOA comes from terrestrial emissions, which dwarf the oceanic benzene and toluene emissions. Hence, adding our calculated mean and maximum Arctic Ocean emission flux to the CAM-Chem model increases SOA concentration by 0.1 and 1.2%, respectively. In contrast, over the pristine Southern Ocean, farther away from continental influence, the impact of oceanic benzene and toluene emission on the SOA pool is far greater: Our cruise-average fluxes increase the total SOA mass concentration by 7.7% and the total organic (primary + secondary) aerosol by 6.7% (Fig. 5). Therefore, ocean biological emissions of benzene and toluene probably contribute to the observed summertime increase in total organic aerosol and SOA over the Southern Ocean (*15*, *57*). In comparison, Arnold *et al.* (*22*) estimated that ocean isoprene emissions contribute only 0.2 to 1.3% to SOA and organic aerosol mass concentrations over the Southern Ocean. Thus, marine benzene and toluene can be more important SOA (and, by extension, organic aerosol) precursors than marine isoprene, at least in the Southern Ocean. Running the model with the cruise-maximum Southern Ocean fluxes instead of the cruise-mean emission fluxes increases the mass concentration of benzene- and toluene-derived SOA by 298.0 and 748.2%, respectively (Fig. 5), compared to no oceanic benzene and toluene emissions. This results in an increase in total SOA of 77.3% and an increase in the total organic aerosol by 67.4% compared to no oceanic benzene and toluene emissions. This indicates that episodes of high ocean emissions of benzene and toluene may have a large local impact on SOA.

The model also estimates the concentration of non–sea-salt sulfate aerosol (derived from oceanic DMS emissions and anthropogenic SO_2_ transport) to be 0.323 μg m^−3^ in the Arctic and 0.059 μg m^−3^ in the Antarctic in all model runs. Comparison with SOA mass concentrations reveals that, despite the addition of marine benzene and toluene emissions to the model, sulfate dominates the secondary aerosol composition in both polar regions. The calculated ocean emission fluxes of DMS were 20-fold those of benzene and toluene during both cruises (*45*, *51*). Consequently, cruise-mean and cruise-maximum benzene and toluene emissions increase the total secondary aerosol (SOA + non–sea-salt sulfate aerosol) by less than 2% in all model runs and for both polar oceans. Although benzene and toluene do not contribute as much to aerosol mass compared to DMS, they are a source of highly oxygenated organic molecules (*58*) that condense onto existing particles and contribute to aerosol growth (*59*). Oxidized aromatics alter particle properties and may help explain the lower-than-expected particle hygroscopicity over the Southern Ocean (*60*). Overall, our modeling results show that ocean benzene and toluene emissions enhance SOA in the polar regions, especially over the pristine Southern Ocean, where episodic localized emissions have a disproportionate contribution to SOA mass.

## DISCUSSION

Here, we present seawater concentrations and atmospheric measurements of benzene and toluene from two cruises in the polar oceans. Benzene and toluene concentrations were measured in surface seawater and in a large number of depth profiles. This unique combination of measurements points toward a biological source for these two compounds previously thought to be predominantly released to the environment from anthropogenic activity. Compared to toluene, benzene appears to correlate stronger with biological variables, such as Chl a or DMS in the underway and depth profile measurements, suggesting that this benzene production may be expressed constitutively by a broad range of phytoplankton. In contrast, the depth profiles and underway concentrations of toluene were less tied to Chl a, suggesting that toluene may be produced by certain groups of phytoplankton or under certain conditions or with the intervention of bacteria (*33*). In the Arctic, benzene and toluene correlated stronger than in the Southern Ocean, suggesting a higher level of anthropogenic pollution that would have masked correlation with other biogenic volatiles. Concurrent high-resolution measurements in the surface seawater and the overlying atmosphere showed that both the Arctic and the Southern Ocean were highly supersaturated in benzene and toluene, and their emission fluxes rivalled those of other atmospherically relevant marine trace gases, such as isoprene or monoterpenes, although the particle yield of benzene and toluene is much higher than that of previously well-researched marine trace gases. Implementing these ocean emission fluxes in a global chemistry-climate model, we estimated that ocean-leaving benzene and toluene made substantial contributions to SOA mass concentration in the polar regions, with the largest effect over the pristine Southern Ocean (7.7% increase in SOA as the average effect and up to 77.3% increase using the highest measured emission flux). Although a note of caution must be sound on the limited spatial and temporal coverage of our cruises, these results indicate that the inclusion of natural oceanic emissions of benzene and toluene in global models will help to reduce the current underestimates in naturally produced total (*16*) and cloud-forming (*18*) aerosols over the oceans. Our findings also call for expanding both the measurements and the model representations of other, hitherto overlooked, gas precursors of SOA in the marine atmosphere.

We note that our modeling focuses on aerosol mass concentrations, whereas benzene and toluene emissions likely also affect aerosol hygroscopicity and thus the ability of aerosol to form clouds. Further studies that track the fate of these aromatic compounds on marine aerosols should be conducted if we are to reduce the uncertainty of climate predictions due to aerosol direct and indirect forcing (*14*). Overall, this work provides previously unknown insights into the connections between polar ocean biological processes and climate through the identification and quantification of previously unidentified natural sources of marine aerosol. This will help to define the preindustrial baseline in aerosol precursors and aid modeling assessments of the subsequent anthropogenic forcing (*19*, *20*).

## MATERIALS AND METHODS

### Benzene and toluene air and seawater measurements and fluxes

Benzene and toluene were measured on both cruises using a PTR-MS (Ionicon PTR-MS, high sensitivity with a quadrupole) coupled to a segmented flow coil equilibrator (SFCE). Details on the installation on board for each cruise are provided elsewhere (*45*, *51*, *61*). Briefly, the PTR-MS and the SFCE were installed on the ship in a laboratory near an underway water tap. During the cruise in the Southern Ocean, an air pump was used to rapidly draw ambient air from about 16 m above sea level to the laboratory, where the PTR-MS subsampled from a tee piece upstream of the pump. Solenoids were used to set up an hourly cycle of measuring SFCE headspace, ambient air, and ambient air passed through a Pt catalyst, which acts as a blank measurement. During the Arctic cruise, SFCE headspace was sampled continuously. The SFCE sampled seawater from the underway water supply (Southern Ocean cruise, depth of 4 to 7 m; Arctic cruise, depth of 3 to 4 m) via an overflowing glass bottle. To make vertical profile measurements, waters were collected gastight from the CTD rosette bottles into 900-cm^3^ glass bottles and sampled with the SFCE [discrete sampling further described in (*51*, *61*)].

In the PTR-MS, *m*/*z* 79 and *m*/*z* 93 (mass-to-charge ratio) were taken to be benzene and toluene, respectively, in accordance with previous mass assignments (*54*, *62*). During both cruises, the PTR-MS was calibrated using a multicomponent gas standard containing benzene and toluene (Supplementary Text). During the Southern Ocean cruise, the hourly measurement of outside air scrubbed by the Pt catalyst was used as a blank for the toluene air measurements, while daily measurements of zero air from a gas standard were used as a blank for benzene air measurements. The air measurements were carefully filtered for ship stack contamination by discarding air measurements collected when the relative wind speed was less than 4 m s^−1^ and the wind was coming from 10° to 70° on either side of the bow. Note that air measurements with the wind blowing from the front are excluded to remove the influence of the foremast vents on our measurements. This led to the removal of a large number of air measurements. To report continuous fluxes, air measurements were interpolated using a smoothing spline interpolation that is shown in Fig. 2.

The ratio between benzene and toluene has been used in the past to spot petroleum combustion plumes from local anthropogenic activity beyond the research/sampling vessel, as in the work of Giorio *et al.* (*29*) (see the Supporting Information thereof). A toluene/benzene ratio of 2 or above is generally considered indicative of fresh fossil fuel burning emissions. Since toluene air mole fractions were generally below the LOD, this ratio was consistently below 1 in our measurements. This supports that we efficiently removed the influence of the sampling vessel on our air measurements and that our air measurements were not substantially affected by other anthropogenic activity, such as fresh fossil fuel burning from nearby commercial ships or scientific research bases.

Dissolved concentrations of benzene and toluene were computed on the basis of solubility from a set of equations presented in the work of Wohl *et al.* (*61*). A detailed discussion of the measurement background and the LOD and measurement uncertainty is provided in the Supplementary Text.

Our specific equations to calculate air-sea fluxes are laid out in detail in the work of Wohl *et al.* (*45*). Here, we provide a brief recap and additional details specific to benzene and toluene. Air-sea fluxes are calculated using the Liss and Slater (*63*) two-layer framework. Benzene and toluene fluxes are computed using the waterside transfer velocity from Nightingale *et al.* (*64*) and the airside transfer velocity from Yang *et al.* (*65*). Freshwater solubilities (dimensionless water over air) for benzene and toluene listed in the work of Wohl *et al.* (*61*) were converted to seawater solubilities at the temperature of ambient seawater as described by Johnson (*66*). The Schmidt number for toluene was calculated using the supplementary R code from Johnson (*66*). The Schmidt number for benzene was also calculated as listed by Johnson (*66*) but assuming the same temperature dependence as toluene. Saturations [see equation 1 in the work of Wohl *et al.* (*45*)] were not computed if the hourly air mole fraction is near or below zero (defined as less than one measurement noise) to avoid unrealistic values. Fluxes were computed even if the hourly air mole fraction is below the LOD. Any filtering of these air mole fractions by setting them to the LOD or zero would artificially bias the reported flux. Calculated fluxes in the Arctic were scaled linearly to the open water fraction as recommended by Prytherch *et al.* (*67*). To do this, the underway SIC was used as derived from the AMSR2 (Advanced Microwave Scanning Radiometer) (*68*).

DMS and isoprene concentrations and fluxes were also measured during both deployments. These data are shown elsewhere (*45*, *51*) and used here to test for correlations with benzene and toluene. We also concurrently measured xylene. However, we refrained from quantifying it and calculating the air-sea exchange because we could not calibrate for xylene using our multicompound gas standard.

### CAM-Chem simulations

We used a global chemistry-climate model, the CAM-Chem [see the work of Lamarque *et al.* (*41*) and Saiz-Lopez *et al.* (*69*)], to quantify the potential effects of the derived oceanic flux of benzene and toluene on atmospheric composition. CAM-Chem is designed to perform both climate simulations (with online simulation of climate system) and simulations with specified dynamics (nudged to offline meteorology fields). The implementation of SOA formation embedded in CAM-Chem is described by Lamarque *et al.* (*41*) and Lack *et al.* (*70*). Briefly, the CAM-Chem model applies the two-product parameterization (*71*) to simulate the SOA formation, considers SOA formations from the oxidation of VOCs (monoterpenes, isoprene, benzene, toluene, and xylene), and includes reactions with key oxidants (OH, O_3_, and NO_3_). The formed quantity of SOA at each time step depends on the gas-phase oxidation rate of VOCs (calculated in the gaseous chemistry module) and the production yield (estimated online based on the existing organic aerosol and the relative abundance of NO versus HO_2_, the so-called high-NO*_x_* and low-NO*_x_* conditions) (*72*). The produced SOA increases the total burden of organic aerosol, either via condensing onto the existing aerosol or forming new aerosols. Here, we further update the model to include alkanes and alkenes as the additional SOA precursors following Mahmud and Barsanti (*73*) and to include Cl and Br atoms as the additional oxidants following Choi *et al.* (*74*) and Li *et al.* (*75*).

The CAM-Chem simulations were conducted in specified dynamic mode (nudged to GEOS5 meteorological data) so that we can isolate the chemical impacts of the additional oceanic emissions of benzene and toluene on the atmospheric levels of benzene, toluene, oxidants (OH), and SOA. The simulations were run from January 2017 to December 2019, and the results in the first 6 months were discarded as spin-up. For the anthropogenic and biomass burning sources of air pollutants, including NO*_x_*, SO_2_, CO, NH_3_, and VOCs (e.g., benzene, toluene, and many others), we used the emission inventories compiled for the ongoing Coupled Model Intercomparison Project 6 (*76*). The terrestrial biological emissions of VOCs (e.g., isoprene and monoterpenes) are calculated online using the Model of Emissions of Gases and Aerosols from Nature 2 (*77*). It is worth mentioning that being a global chemistry-climate model, CAM-Chem includes the transport of terrestrial emissions of VOCs to the marine atmosphere. A climatology of oceanic emission of DMS, used in previous Community Earth System Model simulations [e.g., see the work of Tilmes *et al.* (*78*) and Veres *et al.* (*79*)], is also included, which is the only oceanic emission of reactive gas considered in this model.

In total, we have conducted three simulation cases (table S2), coined the no BT, avg BT, and max BT cases. The no BT case contains no oceanic emissions of benzene and toluene; the avg BT case includes the average oceanic emission fluxes of benzene and toluene for both polar oceans (all oceanic regions >60°N and <−55°S) taken from the cruise measurements; the max BT case applies the maximum oceanic fluxes for the polar oceans (i.e., the highest hourly measured/calculated oceanic outgassing flux). The difference in the atmospheric compositions (benzene and toluene mole fractions, OH, and SOA) between avg BT and no BT cases represents the effects of the average oceanic flux of benzene and toluene in the atmosphere. The difference between max BT and no BT illustrates the impact of the maximum fluxes. Note that all modeling results have been averaged by sampling CAM-Chem grid points at the exact location and time (month) of each cruise. By designing these cases, we attempt to capture the most probable effects of the additional oceanic aromatic emissions (no BT versus avg BT) and the possible range of such effects (no BT versus max BT). The CAM-Chem simulation results are described in the “Atmospheric effects of oceanic benzene and toluene emissions” section.
