# Supplementary material for: Marine biogenic emissions of benzene and toluene and their contribution to secondary organic aerosols over the polar oceans
Source: Sci Adv. 2023 Jan 27;9(4):eadd9031. doi: 10.1126/sciadv.add9031 (PMC9882975; doi:10.1126/sciadv.add9031)
Supplement: Supplementary file 1 — Supplementary Text Figs. S1 to S8 Tables S1 and S2 [file sciadv.add9031_sm.pdf]

Science Advances
AAAS

# Supplementary Materials for

## Marine biogenic emissions of benzene and toluene and their contribution to secondary organic aerosols over the polar oceans

Charel Wohl *et al.*

Corresponding author: Charel Wohl, cwohl@icm.csic.es

*Sci. Adv.* **9**, eadd9031 (2023)
DOI: 10.1126/sciadv.add9031

**This PDF file includes:**

Supplementary Text
Figs. S1 to S8
Tables S1 and S2

## Supplementary Materials

### Supplementary Text

Details on the Chl a data from both cruises

Chl *a* data from both cruises in depth profiles and underway are from fluorescence sensors. The underway Chl *a* data from the Antarctic cruise are relatively uncertain due to large sensor drift, but the data has been corrected using the sensor mounted on the CTD frame. For the Arctic cruise, Chl *a* measured from CTD frame and underway agree within 0.1 mg $m^{-3}$. Thus, our Chl *a* data does not explicitly account for quenching of fluorescence and is not proportional to phytoplankton biomass. It is of value to compare Chl *a* with benzene and toluene distributions in depth profiles and underway data to obtain information on the possible biological source, though it is not possible to derive from this how benzene and toluene concentrations correlate with phytoplankton biomass.

Details on the computation of benzene and toluene seawater and ambient air concentrations as well as fluxes

During the Antarctic cruise, the PTR-MS was calibrated weekly using a certified gas standard (Apel–Riemer Inc.). During the Arctic cruise, benzene was calibrated using a certified gas standard during installation before the campaign. A post-cruise calibration with a certified gas standard was applied to toluene. These calibration curves were used to calculate equilibrator headspace gas phase mole fractions and ambient air mole fractions. During the Arctic cruise, this gas calibration displayed a humidity dependence for benzene and toluene, which was not observed during the Antarctic deployment due to the higher drift tube voltage.

To calculate ambient air measurements, hourly measurement of outside air scrubbed by a 450°C Pt-catalyst is used as a blank for toluene. Daily measurements of zero air carrier gas are used as a blank for benzene. Measurement of zero air from a gas canister are used as a blank for benzene because it seemed that during episodes of high benzene air mole fractions from sampling ship exhaust the Pt-catalyst does not fully remove benzene. For most of the deployment, the Pt-catalyst agrees with the zero air.

For both cruises, benzene and toluene seawater concentrations were calculated using the equations and solubilities laid out in Wohl et al. (*60*). As laid out elsewhere (Supplement of Wohl et al. (*46*) and Wohl et al. (*51*)), the choice of measurement background is critical. Here we lay out what

blanks are used to compute seawater concentrations for both deployments. Please see the supplements of Wohl et al. (*46*) and Wohl et al. (*51*) for detailed definitions of the blanks cited here. For the Antarctic deployment, zero air measurement was used as a blank for benzene seawater measurement. Due to the humidity dependence of the toluene background, we used humid air (air of the same humidity as equilibrator headspace scrubbed by a Pt-catalyst) as a blank for the seawater measurement. Similarly, the Arctic measurements are presented with zero air as a blank for benzene and the wet equilibrator air (zero air which has passed through the empty wet equilibrator) as a blank for toluene. Small differences in the cruise mean concentration presented here and in Wohl et al. (*60*) are due to different choices of background. Concentrations shown here should be cited in the future as they incorporate an improved understanding of the SFCE-PTR-MS system. For both cruises, we thus use zero air as a background for benzene, while toluene blanks were chosen for their similar humidity as equilibrator headspace (wet equilibrator in the Arctic and humid air in the Southern Ocean).

For both cruises, measurements at 5m from CTD casts and proximate underway measurements agree very well suggesting no contamination from either sampling technique. In Wohl et al. (*60*), we discussed that average concentrations of toluene measured from the underway intake system were slightly higher than those measured from the CTD casts. In this manuscript, we display measurements from both sampling techniques which show no obvious bias. It is possible that mean underway concentrations were slightly higher as they by chance captured episodes of higher concentrations. We note that for the Antarctic cruise, benzene and toluene seawater measurements did not appear to be affected by photochemical production within the SFCE.

The measurement noise (Table S1) is calculated as the standard deviation of the residual of the interpolation of the measurement background (as laid out in Wohl et al. (*46*)). The limit of detection is defined as three times the measurement noise. Measurement noise was calculated to be 8 and 10 pmol mol$^{-1}$ for benzene and toluene in the air. The measurement noise in seawater for the Southern Ocean cruise was 1 and 4 pmol dm$^{-3}$ for benzene and toluene while it was 5 and 10 pmol dm$^{-3}$ for benzene and toluene during the Arctic cruise.
The seawater measurement noise is slightly higher in the Arctic compared to the Southern Ocean cruise due to less frequent blanks and different PTR-MS quadrupole data collection settings.

Measurement noise and LOD are low enough to detect these gases in seawater while air mole fractions were often close to the LOD.

**Fig. S1.**

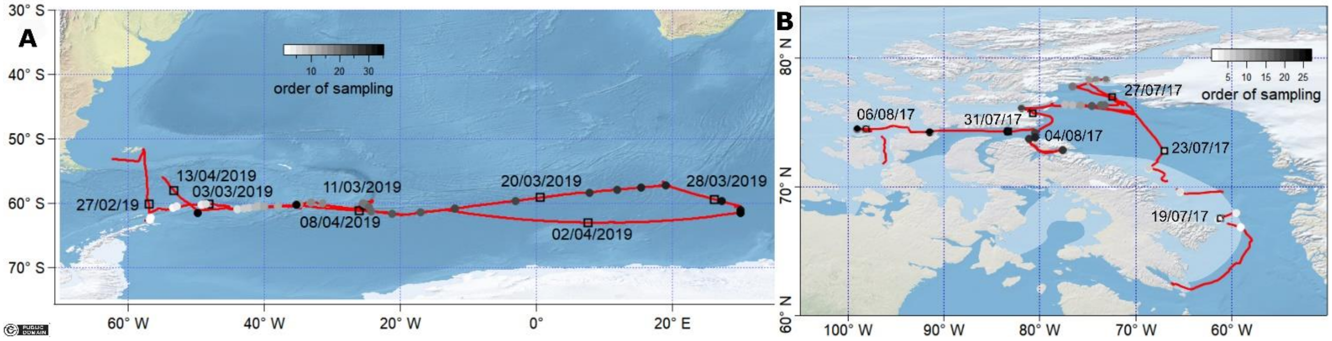


**Figure S1 Map illustrating the cruise sampling tracks in red in the Southern Ocean (A) and the Arctic (B).** Location of the CTD stations is indicated by a filled circle coloured by the order of sampling. Hollow squares and date labels (DD/MM/YYY) are used to give an indication of the sampling date. Interruptions in the cruise track and underway auxiliary data (B) are due to interruptions in the ship underway logging system (*79*). All the map data were created from public domain GIS data found on the Natural Earth website (http://www.naturalearthdata.com, last access: 15 April 2021). They were read into Igor using the Igor GIS XOP beta. The sea-ice-covered area during the Arctic cruise (B) is approximately indicated for illustration purposes as a shaded area due to the dynamic nature of sea ice and difficulties in conveying this information for a month-long deployment. The approximate location of the sea ice edge is based on the average sea ice cover for the whole cruise duration using AMSR2 satellite data.

The cruise sampling tracks of the two cruises in the polar oceans are presented in Figure S1. One of the cruises focussed on sampling the remote Southern Ocean during austral summer and autumn from 21 February to 15 April 2019 on board the RRS *James Clark Ross*. The vessel transited south from the Falkland Islands to the Antarctic Peninsula. For most of the cruise, it then followed approximately 60° S in latitude eastwards, while sampling a large number of CTD (Conductivity/Temperature/Depth) stations, until reaching 30° E. Then the vessel made the return journey and completed a few more CTD stations on its transit back. Essentially no sea ice was encountered during this cruise, only icebergs. The second cruise focused on sampling the marginal sea ice zone in the Canadian Arctic during boreal summer from 17 July until 8 August 2017 on board the icebreaker CCGS *Amundsen*. The research vessel travelled northwards through Davis Straight to Baffin Bay to reach Smith Sound, where more intense depth profile sampling was undertaken. From there the vessel transited east into the Canadian Arctic Archipelago. Further details of the cruise sampling track and strategy are presented in Wohl et al. (*46*, *51*).

**Fig. S2.**

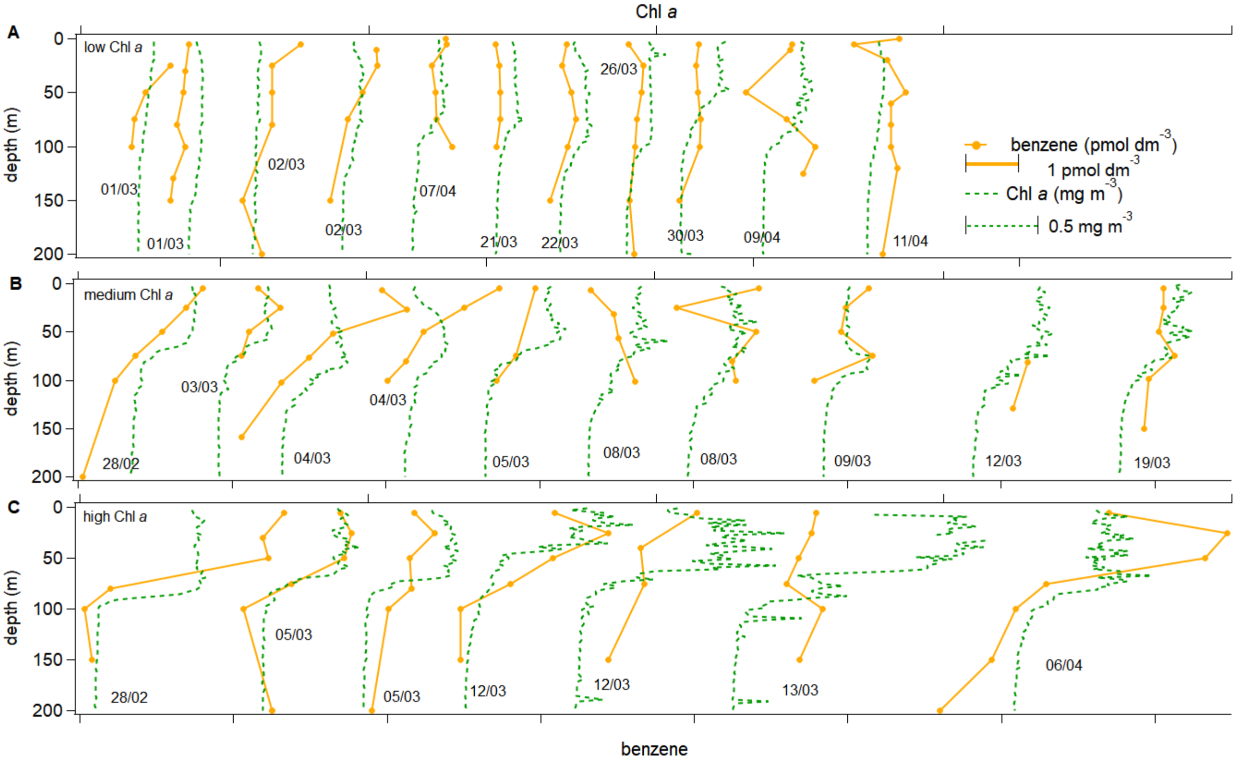


**Figure S2 Overview plot displaying the shape of all benzene and Chl *a* depth profiles from the Southern Ocean**. The casts are grouped in panels by surface Chl *a* concentration (low, medium and high Chl *a*) and staggered along the x-axis for ease of viewing. The scale bars for benzene and Chl *a* in panel (A) also apply to panels (B) and (C). Date labels indicate sampling dates (DD/MM).

**Fig. S3.**

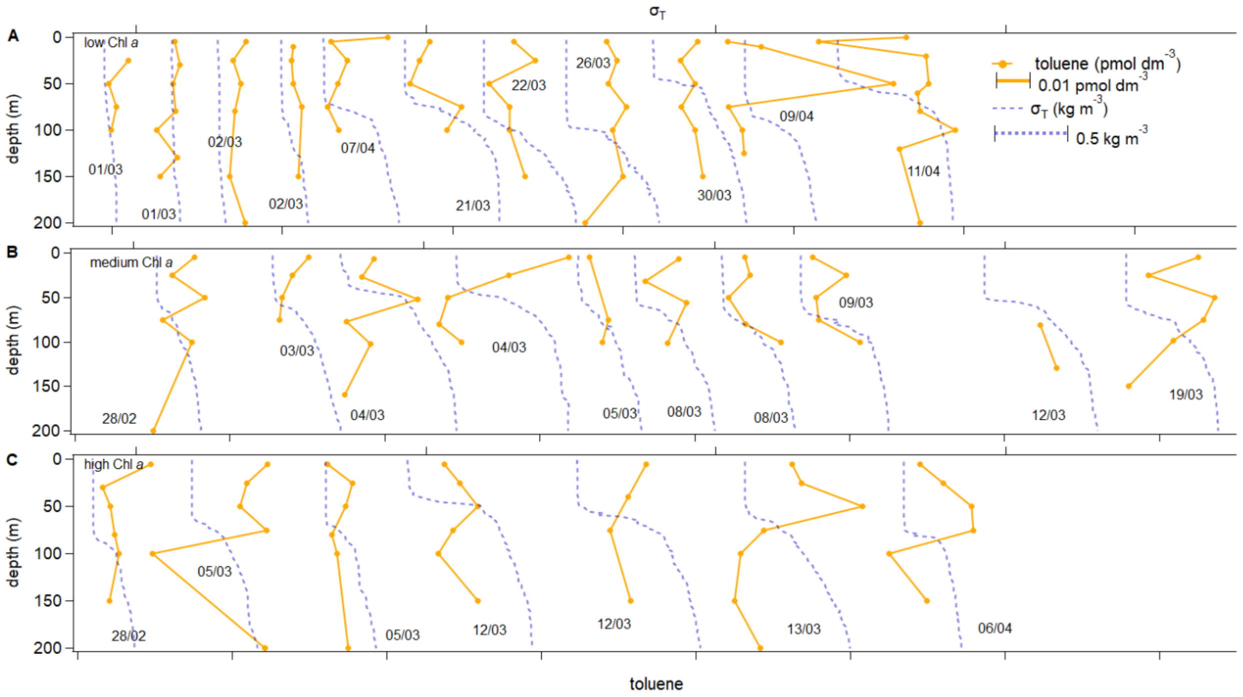


**Figure S3 Overview plot displaying the shape of all toluene and density depth profiles from the Southern Ocean**. The casts are grouped in panels by surface Chl a concentration (low, medium and high Chl a) and staggered along the x-axis for ease of viewing. The scale bars for toluene and Chl a in panel (A) also apply to panels (B) and (C). Date labels indicate sampling dates (DD/MM).

**Fig. S4.**

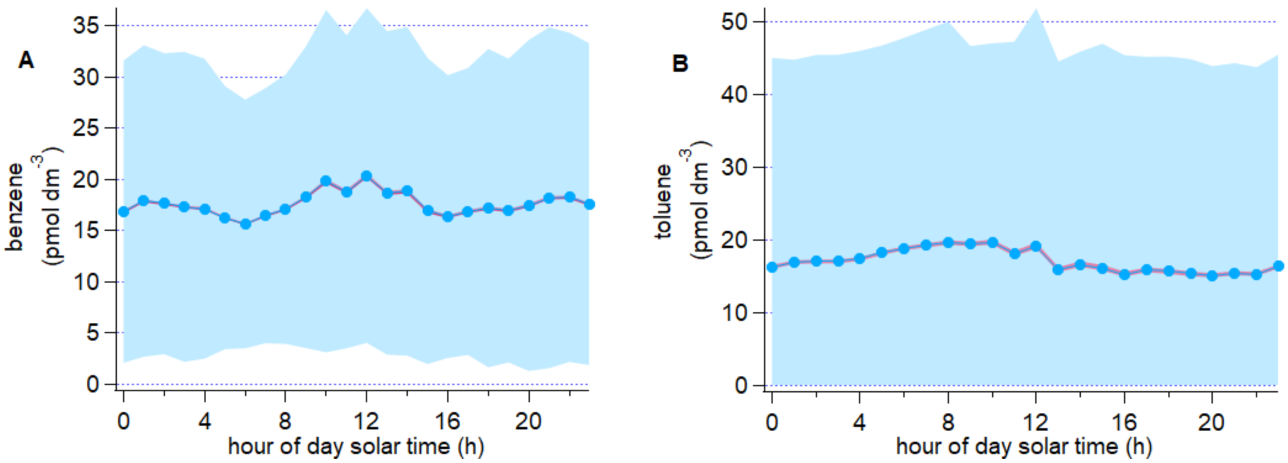


**Figure S4 Diurnal changes in seawater concentrations of (A) benzene and (B) toluene in underway surface seawater**. Light blue shaded area indicates the standard deviation of each hourly bin and the pink shaded area indicates the standard error of each bin.

We test for diurnal variability in the measured seawater concentrations from the Southern Ocean by using the local solar time to remove the influence of the ship track crossing multiple time zones. The measured seawater concentrations were binned in 24 hourly bins and the standard deviation and standard error was calculated for each bin (Figure S2). Figure S2 shows that measurements of benzene and toluene during solar zenith are generally a little higher than the other measurements, but there is no substantial diurnal variability in seawater benzene and toluene concentrations and this variability is not statistically significant. The Arctic data was not tested for diurnal variability due to 24 h light during the sampling period and the relatively smaller dumber of data points compared to the Southern Ocean cruise.

**Fig. S5.**

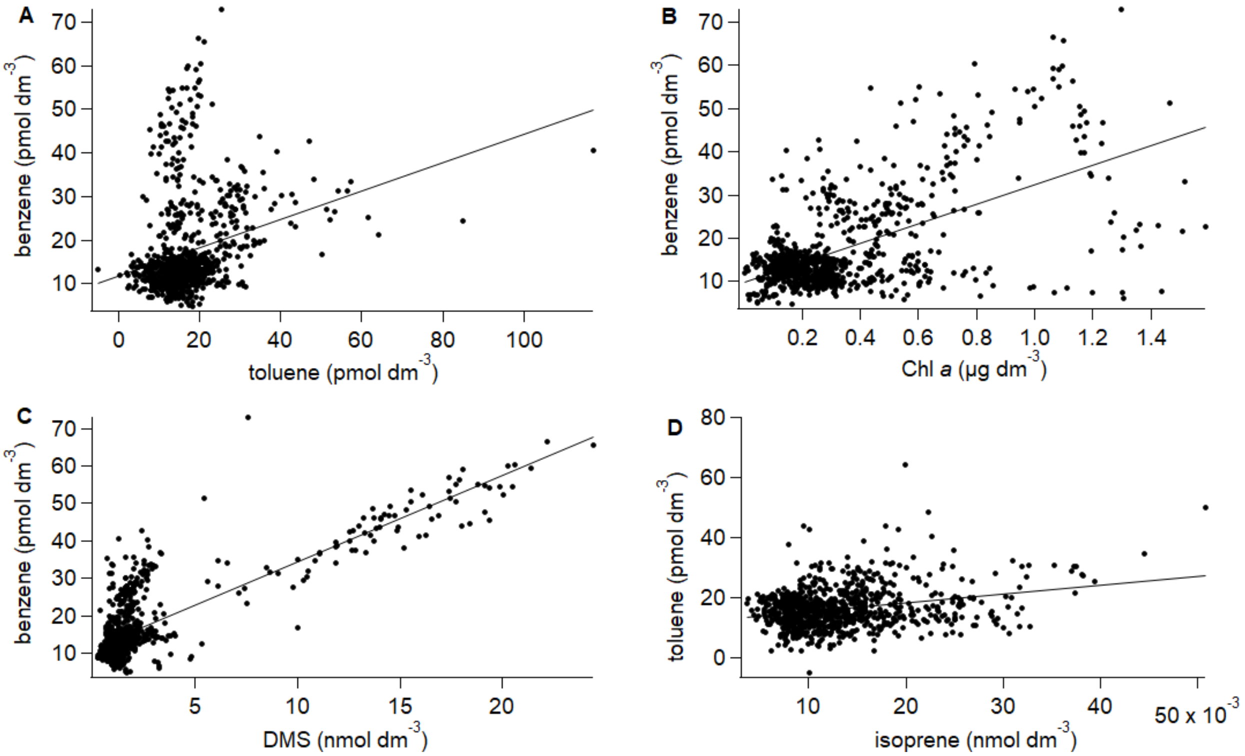


**Figure S5 Scatter plot of the correlations between underway seawater measurements of benzene, toluene and chlorophyll *a*, as well as DMS and isoprene, from the Southern Ocean cruise.** Details on the statistics related to these correlations are stated in the main text.

## Fig. S6.

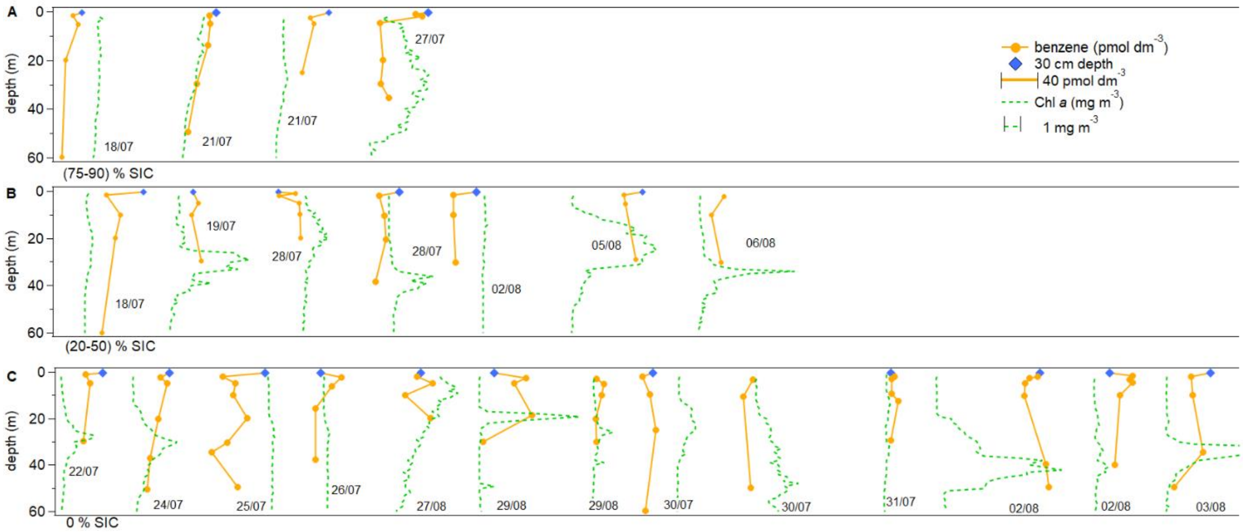


**Figure S6 Overview plot displaying the shape of all benzene and Chl *a* depth profiles from the Arctic cruise, grouped by SIC.** Panel labels indicate the SIC bin. The scale bars for benzene and Chl *a* in panel (A) apply also to panels (B) and (C). One of the Chl *a* profiles is cut off in panel (C) for scale purposes.

## Fig. S7.

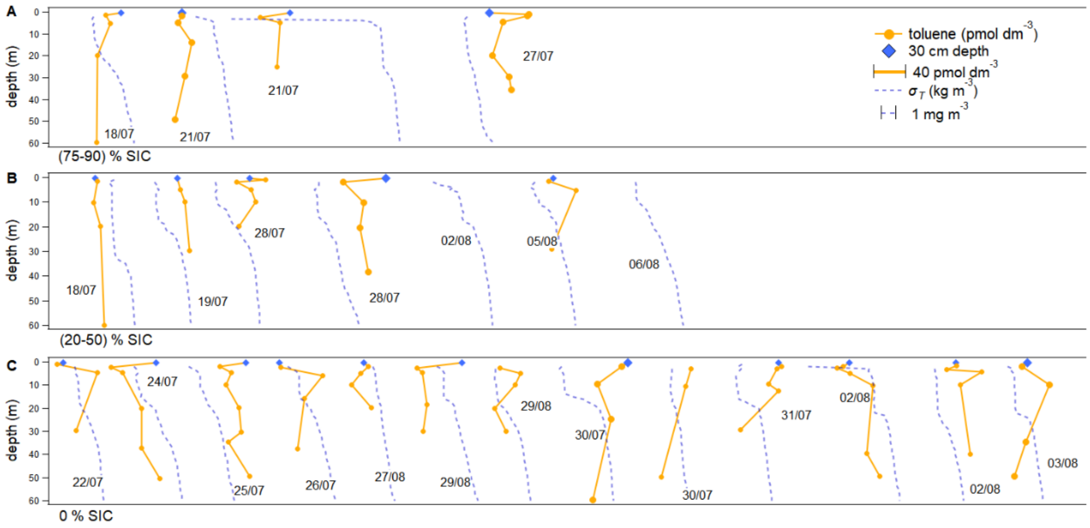


**Figure S7 Overview plot displaying the shape of all toluene and density depth profiles from the Arctic cruise, grouped by SIC.** Panel labels indicate the SIC bin. The scale bars for toluene and density in panel (A) apply also to panels (B) and (C).

**Fig. S8.**

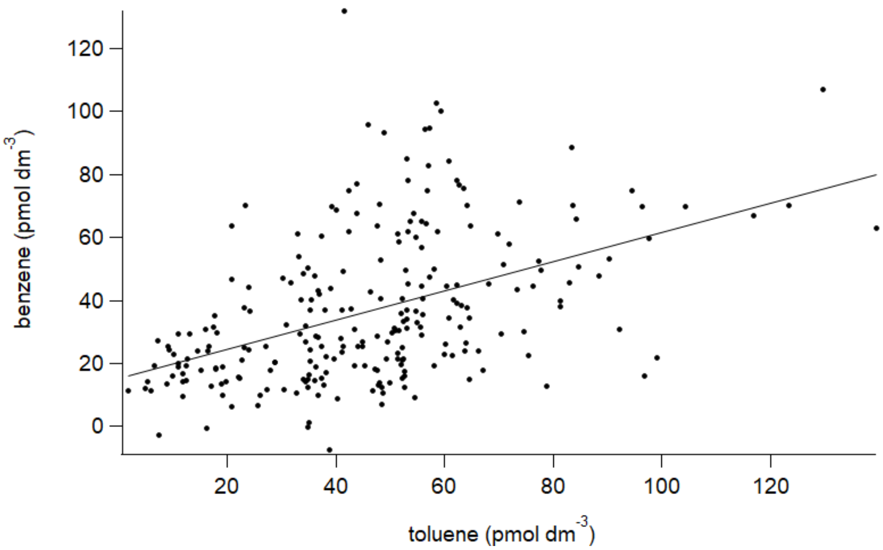


**Figure S9 Scatter plot of the correlation between the underway seawater measurements of benzene and toluene from the Arctic cruise.** Details on the statistics related to this correlations are stated in the main text.

**Table S1.**

**Table S1 Fluxes and saturations calculated for the Arctic deployment using higher air mole fractions as modelled by Cabrera-Perez et al.** (*25*)**.** For this calculation, all other parameters were kept the same and only the air mole fractions were increased to the values indicated in the table.

| | benzene | toluene |
|---|---|---|
| Air mole fraction (pmol $mol^{-1}$) | 50 | 30 |
| Saturation (%) | 159 | 311 |
| Flux (µmol $m^{-2}$ $d^{-1}$) | 0.011 | 0.025 |

Table S1 shows that using higher air mole fractions in the Arctic does not change the conclusion of oceanic outgassing of benzene and toluene in the Arctic. Using higher air mole fractions, decreases the estimated benzene saturation by 74 % and the flux by 52 %. Similarly, using higher toluene mole fractions decreases the saturation by 87 % and the flux by 26 %. This calculation gives an appreciation of the potential uncertainty of the fluxes reported for the Arctic deployment due to the choice in air mole fraction.

## Table S2.

## Table S2 CAM-Chem simulation cases

| Cases | Oceanic emission of Benzene in Polar regions | Oceanic emission of Toluene in Polar regions |
|---|---|---|
| **noBT** | - | - |
| **avgBT** | Arctic, >60ºN: 0.0244 μmol $m^{-2}$ $d^{-1}$<br>Antarctic, <-55ºS: 0.0236 μmol $m^{-2}$ $d^{-1}$ | Arctic, >60ºN: 0.0341 μmol $m^{-2}$ $d^{-1}$<br>Antarctic, <-55ºS: 0.0390 μmol $m^{-2}$ $d^{-1}$ |
| **maxBT** | Arctic, >60ºN: 0.158 μmol $m^{-2}$ $d^{-1}$<br>Antarctic, <-55ºS: 0.358 μmol $m^{-2}$ $d^{-1}$ | Arctic, >60ºN: 0.268 μmol $m^{-2}$ $d^{-1}$<br>Antarctic, <-55ºS: 0.158 μmol $m^{-2}$ $d^{-1}$ |
